# Satisfying medical and rehabilitation needs positively influences returning to work after a work-related injury: an analysis of national panel data from 2018 to 2019

**DOI:** 10.1186/s12889-021-12064-1

**Published:** 2021-11-05

**Authors:** Suk Won Bae, Min-Yong Lee, Shin Who Park, Gangpyo Lee, Ja-Ho Leigh

**Affiliations:** 1 Department of Rehabilitation Medicine, Seoul National University Hospital, Seoul, 03080 Korea; 2 Rehabilitation Medical Center, Korea Workers’ Compensation and Welfare Service Incheon Hospital, Incheon, 21417 Korea; 3 Rehabilitation Medicine Research Center, Korea Workers’ Compensation and Welfare Service Incheon Hospital, Incheon, 21417 Korea; 4 National Traffic Injury Rehabilitation Research Institute, National Traffic Injury Rehabilitation Hospital, Yangpyeong, 12564 Korea

**Keywords:** Medical needs, Rehabilitation needs, Return to work, Work-related injury, Panel study of workers’ compensation insurance

## Abstract

**Background:**

This study examined how meeting the medical needs of injured workers after initial treatment may affect their return to work, using data from the Panel Study of Workers’ Compensation Insurance.

**Methods:**

This study was designed as a longitudinal study, which used data from the second-year, follow-up survey conducted in the secondary cohort of the Panel Study of Workers’ Compensation Insurance. The odds ratio (OR) and 95% confidence interval were estimated through binomial and multinomial logistic regression analyses to examine the effects of unmet medical needs on workers’ return to original work and return to work overall (including reemployment).

**Results:**

The returned to original work OR of workers whose rehabilitation needs were met was 1.35 (1.12–1.63) while the return to work OR was 1.20 (1.03–1.41). The returned to original work OR of workers whose medical needs were met was 1.64 (1.18–2.27) while the return to work OR was 1.39 (1.07–1.80). In terms of disability rating, the return to work ORs of workers with mild disabilities whose medical/rehabilitation needs were not met and those of workers without disabilities were 1.71 (1.17–2.49) and 1.97 (1.27–3.08), respectively. In the case of regular/temporary workers, the returned-to-work ORs of workers whose medical/rehabilitation needs were not met were 1.54 (1.12–2.13) and 1.27 (1.03–1.56), respectively.

**Conclusions:**

For workers who sustained work-related injuries, providing medical accessibility and meeting rehabilitation needs were found to be important predictors of return to work after initial treatment.

**Supplementary Information:**

The online version contains supplementary material available at 10.1186/s12889-021-12064-1.

## Introduction

Health and health equality are important goals for the development of a society [1, 2]. Socioeconomic status directly impacts individuals’ health and can lead to health inequality [3, 4]. Specifically, a low socioeconomic status can deteriorate health [5], which can lead to an unbalanced use of medical services, thus becoming a vicious cycle that worsens health inequality [6, 7].

To maintain health, it is vital that individuals avail themselves of medical services when they are deemed necessary [8]. The status of individuals’ unmet medical needs is considered an indicator for measuring accessibility to medical services [9, 10]. Unmet medical needs were shown to be high among workers with long weekly working hours [2, 11], low-income workers, and older adult workers [12, 13]. Along with these individual factors, regional factors, such as the degree of urbanization or share of private hospital beds in an area, may also influence unmet medical needs [14, 15]. The few studies that examined the unmet medical needs of workers who sustained work-related injuries reported that workers with relatively low socioeconomic status [4, 15] and workers with disabilities experienced unmet medical needs more frequently [16]. However, few studies have examined the relationship between unmet medical needs and topics related to injured workers’ return to work.

To encourage the use of medical services and stimulate compensation for work-related injuries, the Government of South Korea has continuously expanded the national insurance coverage for such cases. According to the Industrial Accident Data collected by the South Korean Ministry of Employment and Labor, the number of workers who sustained work-related injuries was 109,242 in 2019 [17]. Although the number of injured workers had been decreasing in the 10 years prior to 2018, it has spiked since 2018 [18, 19]. This is attributable to policies that strengthened the accessibility and coverage of the Industrial Accident Compensation Insurance, rather than to an increase in the number of work-related injuries. Accessibility to the insurance was fortified through the Industrial Accident Compensation Insurance Act, which recognizes injuries that occur during the commute to and from work as work-related injuries. Additionally, by abolishing the verification system of business owners, workers can now apply for workplace injury compensation without the employer’s confirmation [20]. Regarding insurance coverage, the use of medical and rehabilitation services by injured workers is supposed to encourage their return to society and work [15, 21]. In fact, the annual rate of return to original work (including reemployment) increased from 50.1% in 2011 to 68.5% in 2019 [22]. Therefore, it is evident that both strengthening accessibility to and coverage of insurance are related to returning to work.

One of the goals of the Industrial Accident Compensation Insurance Act is to ensure that injured workers return to work [23], and numerous studies have examined the factors affecting this return [24]. These factors include gender/sex; age; marital status; educational level; household income; national (professional engineer, engineer, master craftsman, industrial engineer, craftsman, other national certificates), private, and international (foreign-country issued—with an exception of the driver’s license) certifications; number of employees in the company/duration of employment/work status at the time of work-related injury; disability rating after a work-related injury; and periods of hospitalization and recovery [25–31].

Numerous studies have separately analyzed work-related injuries, unmet medical needs [4, 15, 16], and return to work [25–31]. However, few studies have examined the association between unmet medical needs and return to work. Therefore, this study aimed to examine how meeting the medical needs of injured workers after initial treatment may affect their return to work, using data from the Panel Study of Workers’ Compensation Insurance.

## Methods

### Study design and participants

This study used data from the second-year, follow-up survey conducted in the secondary cohort of the Panel Study of Workers’ Compensation Insurance, provided by the Korea Workers’ Compensation and Welfare Service. The aforementioned panel study was conducted to investigate the life of workers after the period of compensation, including return to work and unmet medical needs, and to prepare baseline data for supporting such examinations. The target population of the study was 75,392 injured workers whose claims were closed in 2017. Of those, 3294 were selected for the sample via stratified systematic sampling, with injury classification, sex, and age as the stratification variables. The survey has been conducted annually (between August and October) since 2018; currently, though the third wave survey was completed, the data were unavailable. Thus, the second wave data were analyzed in this study. The panel study used the survey method of Tablet Assisted Personal Interviewing.

**Fig. 1:**
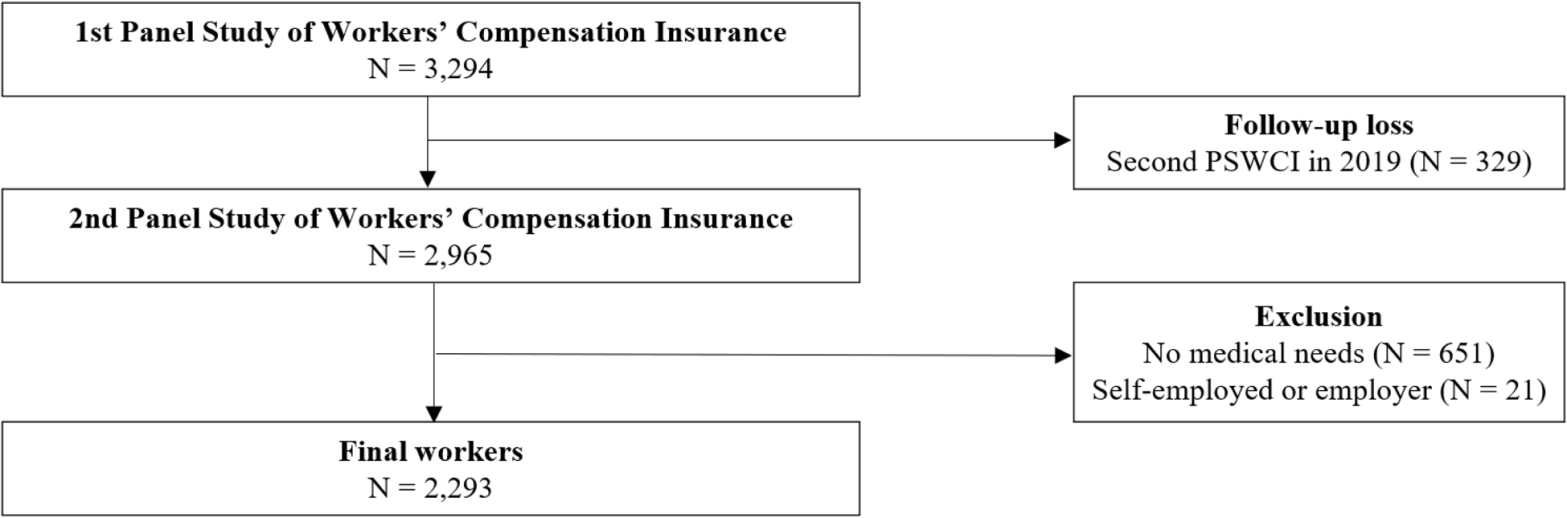
Schematic diagram depicting the study population

The findings from the second-year, follow-up survey showed that 2965 workers from the original sample (i.e., 3294 workers) were retained, indicating a sample retention rate of 90.0%. Only those who consecutively participated in both survey rounds were considered as participants for the current study; accordingly, 329 workers who failed to meet this requirement were excluded. Moreover, 651 workers did not have any medical needs and 21 workers had a status of “self-employed” or “employed” at their place of work during the occurrence of the work-related injury and were therefore excluded. Workers whose work status was either “self-employed” or “employer” at the time of the occurrence of a work-related injury would have the same worker status—namely, “self-employed” or “employer”—when returning to their original work. Accordingly, the data of workers with the “self-employed” or “employer” status were dropped from the analysis. In total, data from 2293 workers were included in the analysis. Figure 1 presents a schematic depiction of the study population.

### Main outcome variables

Six types of economic activity were surveyed in the Panel Study of Workers’ Compensation Insurance: return to original work, reemployed, self-employed, unpaid family work, unemployed, and economic inactivity. In this study, they were recategorized to describe the return to work variable as follows: return to original work; being reemployed or self-employed were categorized as “reemployed”; and unpaid family work, unemployed or economic inactivity were categorized as “non return to work.”

In the survey, the workers’ employment status was investigated yearly. Return to work was defined as the first return to work outcome of paid work (excluding self-employment) across the two wave surveys. Specifically, when participants responded “return to original work” or “reemployed” in the first survey, but then switched to “non return to work” in the subsequent survey, they were classified as either “return to original work” or “reemployed.” Further, when workers responded “unemployed” or “economic inactivity” in the first survey, but then switched to “return to original work” or “reemployed” in the subsequent survey, they were classified as one of the latter two [32]. The details of the procedures and categories of the return to work status are described in Supplementary Table 1.

### Sociodemographic characteristics

The participants’ ages were categorized as follows: less than 40, 40–49, 50–59, and 60 and above. Marital status was categorized into the following: not married, married, and other (i.e., separated, divorced, or widowed). Educational level was categorized as: less than high school, high school, and college or above. The Government of South Korea divides regions according to location and population ratio. Correspondingly, regions were categorized as either Seoul/Gangwon, Gyeongin, Chungcheong/Sejong/Daejeon, Gwangju/Jeolla, Daegu/Gyeongbuk, and Busan/Ulsan/Gyeongnam.

### Employment characteristics

Employment characteristics included the industry type and the work status of participants at the workplace where they sustained the work-related injury. For industry variables, this study followed the 10th Korean Standard Industrial Classification, which is based on the International Standard Industrial Classification; the categories were manufacturing, construction, service, and other.

Regarding work status, regular workers were those with an employment contract of over 1 year and who received various benefits (e.g., bonuses and severance pay); temporary workers were those with an employment contract of over 1 month and less than a year, and who were recruited to fulfill the needs of a specific project; and daily workers were those employed on a daily basis and who received a daily wage. Further, work status was classified into either regular/temporary worker or daily worker.

### Injury-related characteristics

The types of work-related injuries were organized into the following: injury, disease, and commuting injury. The recovery period was categorized as: 3 months or less, 4 to 6 months, 7 to 9 months, and 10 months or more. Disability ratings were classified into 14 categories as stipulated in Korea’s Industrial Accident Compensations Insurance Act; in this stipulation, the severity of damage or disability increases with lower ratings (i.e., Grade 1 for most severe; Grade 14 for least severe) [25], being categorized into the following: 1–7 as severe, 8–11 as moderate, 12–14 as mild, and none.

### Unmet medical needs characteristics

Depending on participants’ answers to the question of whether the given treatment period was appropriate for treating the injured area (i.e., appropriate treatment period), responses were categorized into the following: yes and no.

Depending on whether participants needed support for recurrence prevention and health promotion (i.e., rehabilitation service needs—they either needed or did not need support for complication prevention and exercise), responses were categorized into “yes” and “no.”

Participants were also asked, “Did you experience any medical needs in the past year that required attention (e.g., treatment or examination at medical facilities) but that did not receive attention?” Responding “yes” indicated that they had unmet medical needs, and “no” meant otherwise. Data from workers who responded that they had never needed medical treatment or examination were excluded.

### Statistical analyses

We conducted a chi-squared test to determine the characteristics of participants’ return to work. The odds ratio (OR) and 95% confidence interval were estimated through binomial and multinomial logistic regression analyses to examine the effects of unmet medical needs on workers’ return to original work, and return to work overall (including reemployment). Models were also stratified by disability rating, industry, and status of workers. In the multinomial logistic regression model, the “non return to work group,” the “returned to original work group,” and the “reemployed group” were compared. In the binomial logistic regression model, the “returned to original work group” and “reemployed group” were combined to form a “return to work group.” We used “non return to work” groups as the reference category for both binomial and multinomial logistic regression models. All analyses were conducted using SAS statistical package version 9.4 (SAS Institute, Cary, NC, USA).

## Results

Participants’ characteristics regarding return to work status are summarized in Table 1. Be included below Table 1 that row percentages are shown in the table. For workers below the age of 40, the rate of return to original work was 38.4% and the rate of reemployment was 44.4%; for workers over the age of 60, the rate of non return to work was 45.8% (*p* < 0.001). The rate of return to original work for men was 30.1%, which was slightly higher than that for women (28.4%; *p* = 0.732). The corresponding rate for married workers was 33.0%, which was significantly higher compared to that for single workers (27.8%; *p* < 0.001). Regarding industries, the rate of manufacturing workers who returned to their original work was 42.3%; for construction workers, the rate of those who were reemployed and categorized as non return to work was 52.5 and 35.0%, respectively, exhibiting a higher rate for non return to work than workers in other industries (*p* < 0.001).

**Table 1:** Participants’ characteristics by return to work status

Variables	Non return to work		Returned to original work		Reemployed		*P*-value *
	N	%	N	%	N	%	
Total	662	28.9	683	29.8	948	41.3	
Age (years)							< 0.001
< 40	52	17.2	116	38.4	134	44.4	
40–49	76	17.2	171	38.6	196	44.2	
50–59	205	24.7	263	31.7	362	43.6	
≥60	329	45.8	133	18.5	256	35.7	
Sex							0.732
Male	538	28.7	561	30.1	764	41.0	
Female	124	28.8	122	28.4	184	42.8	
Marital status							< 0.001
Not married	96	28.7	93	27.8	146	43.6	
Married	436	27.9	515	33.0	611	39.1	
Other	130	32.8	75	18.9	191	48.2	
Educational level							< 0.001
Less than high school	358	39.3	185	20.3	367	40.3	
High school	249	24.3	355	34.6	421	41.1	
College or above	55	15.4	143	39.9	160	44.7	
Area							0.001
Seoul/Gangwon	103	28.5	88	24.4	170	47.1	
Gyeongin	182	27.0	209	31.0	283	42.0	
Chungcheong/Sejong/Daejeon	68	27.1	63	25.1	120	47.8	
Gwangju/Jeolla	68	26.5	78	30.4	111	43.2	
Daegu/Gyeongbuk	71	27.1	96	36.6	95	36.3	
Busan/Ulsan/Gyeongnam	170	34.8	149	30.5	169	34.6	
Industry							< 0.001
Manufacturing	170	24.1	299	42.3	238	33.7	
Construction	265	35.0	95	12.5	398	52.5	
Service	92	29.1	107	33.9	117	37.0	
Other	135	26.4	182	35.6	195	38.1	
Work status							< 0.001
Regular/Temporary worker	377	24.6	598	39.1	555	36.3	
Daily worker	285	37.4	85	11.1	393	51.5	
Type of work-related injury							< 0.001
Injury	592	27.8	630	29.5	911	42.7	
Disease	64	43.8	51	34.9	31	21.2	
Commuting injury	6	42.9	2	14.3	6	42.9	
Recovery period, in months							< 0.001
≤3	54	14.2	164	43.0	163	42.8	
4–6	173	20.9	261	31.6	392	47.5	
7–9	126	24.7	147	28.8	237	46.5	
≥10	309	53.7	111	19.3	156	27.1	
Disability rating							< 0.001
Severe	185	66.1	46	16.4	49	17.5	
Moderate	180	31.3	161	28.0	234	40.7	
Mild	225	22.1	308	30.3	485	47.6	
None	72	17.1	168	40.0	180	42.9	
Treatment period appropriate							< 0.001
Yes	189	22.5	319	38.0	331	39.5	
No	473	32.5	364	25.0	617	42.4	
Rehabilitation service needs							< 0.001
Yes	504	32.1	444	28.3	623	39.7	
No	158	21.9	239	33.1	325	45.0	
Unmet medical needs							0.113
Yes	61	33.0	43	23.2	81	43.8	
No	601	28.5	640	30.4	867	41.1	

*Analyses were done by chi-square test

Regarding occupational characteristics, the rate of regular/temporary workers who returned to their original work was 39.1%, and that of daily workers who were reemployed was 51.5% (*p* < 0.001). Regarding disability ratings, the rate of workers with severe disabilities for non return to work was 66.1%, and the rate of those without work-injury-related disabilities who returned to their original work was 40.0% (*p* < 0.001). The rate of return to original work of workers who deemed their treatment period as sufficient was higher than that of their counterparts (*p* < 0.001); the same occurred for those who deemed their rehabilitation needs to have been met, and who had higher rates for return to original work than their counterparts (*p* < 0.001).

The return to work ORs by unmet medical needs are presented in Table 2. The ORs for return to original work and for return to work in workers who deemed their treatment period as sufficient were 1.72 (1.34–2.21) and 1.41 (1.14–1.76), respectively. The OR for return to original work in workers who deemed their rehabilitation needs to have been met was 1.35 (1.12–1.63), while the OR for return to work was 1.20 (1.03–1.41). Further, the OR for return to original work in workers who deemed their medical needs to have been met was 1.64 (1.18–2.27), while the OR for return to work was 1.39 (1.07–1.80).

**Table 2:** Odds ratio of return to work status by treatment period, rehabilitation service needs, and unmet medical needs

	Binomial^a^		Multinomial^b^			
	Return to work		Returned to original work		Reemployed	
	OR^c^	95% CI	OR	95% CI	OR	95% CI
Treatment period appropriate						
Yes	1.41	1.14–1.76	1.72	1.34–2.21	1.24	0.98–1.56
No	1.00		1.00		1.00	
Rehabilitation service needs						
Yes	1.00		1.00		1.00	
No	1.20	1.03–1.41	1.35	1.12–1.63	1.14	0.96–1.35
Unmet medical needs						
Yes	1.00		1.00		1.00	
No	1.39	1.07–1.80	1.64	1.18–2.27	1.26	0.95–1.66

^a^The returned to original work group and the reemployed group were integrated into a single return to work group

^b^The comparisons among the non return to work group and the returned to original work group and reemployed group were conducted separately

^c^The statistical estimations based on the binomial and multinomial multivariate logistic regression analyses were adjusted for all covariates, except for the explanatory variable of interest

Tables 3 and 4 show the ORs of return to work for unmet medical needs and rehabilitation service needs, respectively, stratified with the disability rating, industry, and status of workers. In the case with no medical needs, the OR of return to work for those with a disability rating of mild disabilities was 1.71 (1.17–2.49). By industry, the OR was 2.84 (1.18–6.84) for service workers and 1.54 (1.12–2.13) for regular/temporary workers.

**Table 3:** Odds ratios of return to work status by unmet medical needs (stratified disability ratings, industry, and work status)

		Binomial^a^		Multinomial^b^			
		Return to work		Returned to original work		Reemployed	
		OR^c^	95% CI	OR^c^	95% CI	OR^c^	95% CI
Disability rating	Severe						
	Unmet medical needs						
	Yes	1.00		1.00		1.00	
	No	0.56	0.26–1.21	1.08	0.32–3.67	0.43	0.18–0.99
	Moderate						
	Unmet medical needs						
	Yes	1.00		1.00		1.00	
	No	1.52	0.95–2.42	1.76	0.94–3.28	1.41	0.85–2.34
	Mild						
	Unmet medical needs						
	Yes	1.00		1.00		1.00	
	No	1.71	1.17–2.49	1.68	1.05–2.69	1.67	1.12–2.50
	None						
	Unmet medical needs						
	Yes	1.00		1.00		1.00	
	No	1.29	0.58–2.84	2.05	0.80–5.26	1.00	0.44–2.29
Industry	Manufacturing						
	Unmet medical needs						
	Yes	1.00		1.00		1.00	
	No	1.45	0.90–2.35	1.42	0.83–2.42	1.47	0.85–2.52
	Construction						
	Unmet medical needs						
	Yes	1.00		1.00		1.00	
	No	1.44	0.95–2.20	1.59	0.82–3.05	1.41	0.91–2.19
	Service						
	Unmet medical needs						
	Yes	1.00		1.00		1.00	
	No	2.84	1.18–6.84	3.51	1.15–10.70	2.46	0.95–6.34
	Other						
	Unmet medical needs						
	Yes	1.00		1.00		1.00	
	No	1.00	0.57–1.77	1.48	0.73–2.98	0.76	0.41–1.39
Work status	Regular/temporary worker						
	Unmet medical needs						
	Yes	1.00		1.00		1.00	
	No	1.54	1.12–2.13	1.85	1.27–2.69	1.35	0.96–1.92
	Daily worker						
	Unmet medical needs						
	Yes	1.00		1.00		1.00	
	No	1.14	0.73–1.78	1.13	0.57–2.24	1.15	0.72–1.84

^a^The returned to original work group and the reemployed group were integrated into a single return to work group

^b^The comparisons among the non return to work group and the returned to original work group and reemployed group were conducted separately

^c^The statistical estimations based on the binomial and multinomial multivariate logistic regression analyses were adjusted for all covariates, except for the explanatory variable of interest

**Table 4:** Odds ratios of return to work status by rehabilitation service needs (stratified disability ratings, industry, and work status)

		Binomial^a^		Multinomial^b^			
		Return to work		Returned to original work		Reemployed	
		OR^c^	95% CI	OR^c^	95% CI	OR^c^	95% CI
Disability rating	Severe						
	Rehabilitation service needs						
	Yes	1.00		1.00		1.00	
	No	1.21	0.72–2.03	1.71	0.89–3.29	0.88	0.46–1.68
	Moderate						
	Rehabilitation service needs						
	Yes	1.00		1.00		1.00	
	No	0.96	0.71–1.29	1.23	0.85–1.78	0.82	0.59–1.14
	Mild						
	Rehabilitation service needs						
	Yes	1.00		1.00		1.00	
	No	1.11	0.88–1.41	1.18	0.88–1.57	1.10	0.86–1.41
	None						
	Rehabilitation service needs						
	Yes	1.00		1.00		1.00	
	No	1.97	1.27–3.08	2.00	1.23–3.24	1.97	1.24–3.14
Industry	Manufacturing						
	Rehabilitation service needs						
	Yes	1.00		1.00		1.00	
	No	0.94	0.70–1.28	0.99	0.71–1.38	0.90	0.64–1.26
	Construction						
	Rehabilitation service needs						
	Yes	1.00		1.00		1.00	
	No	1.12	0.86–1.45	1.46	0.99–2.15	1.05	0.80–1.38
	Service						
	Rehabilitation service needs						
	Yes	1.00		1.00		1.00	
	No	1.35	0.84–2.16	2.08	1.21–3.59	1.03	0.62–1.71
	Other						
	Rehabilitation service needs						
	Yes	1.00		1.00		1.00	
	No	1.76	1.23–2.53	1.81	1.21–2.69	1.75	1.18–2.58
Work status	Regular/temporary worker						
	Rehabilitation service needs						
	Yes	1.00		1.00		1.00	
	No	1.27	1.03–1.56	1.39	1.11–1.75	1.16	0.93–1.45
	Daily worker						
	Rehabilitation service needs						
	Yes	1.00		1.00		1.00	
	No	1.14	0.88–1.47	1.35	0.90–2.01	1.10	0.84–1.44

^a^The returned to original work group and the reemployed group were integrated into a single return to work group

^b^The comparisons among the non return to work group and the returned to original work group and reemployed group were conducted separately

^c^The statistical estimations based on the binomial and multinomial multivariate logistic regression analyses were adjusted for all covariates, except for the explanatory variable of interest

In the case with no rehabilitation needs, the OR of return to work in workers with a disability rating of no disabilities was 1.97 (1.27–3.08). By industry, the OR was 1.76 (1.23–2.53) for other types of workers and 1.27 (1.03–1.56) for regular/temporary workers.

## Discussion

According to participants’ return to work status (as shown in Table 1), those who were younger, male, married, and had a higher educational level displayed a higher rate for return to work. According to occupational characteristics, the rate for return to work was higher in those who worked in the manufacturing industry as regular workers during the occurrence of the work-related injury. According to injury-related characteristics, those with a shorter recovery period, with a higher numerical disability rating, and without a disability demonstrated a higher rate for return to work. These findings are similar to the results of previous studies on the return to work of injured workers [26–31]. Specifically, the present findings indicated that, compared with workers in the manufacturing and service industries, those in the construction industry displayed a lower rate for return to original work and a higher rate of reemployment after the work-related injury. This may be because of the characteristics inherent to the construction industry, such as the higher share of daily workers [33] and a longer recovery period than that found in the other two industries [34].

Regarding return to work for unmet medical needs, the OR for return to original work of workers who deemed their treatment period sufficient was 1.72 (1.34–2.21); that for those who deemed their rehabilitation service needs to have been met was 1.35 (1.12–1.63); and that for those who deemed their medical needs to have been met was 1.64 (1.18–2.27) (Table 2). These findings concur with the results of precedent studies, which showed that better perceived health by workers led to higher rates for return to original work [4, 15] and that medical or rehabilitation needs influenced injured workers’ decisions to return to work.

The OR for return to work was high in workers with mild disabilities and those without disabilities (Tables 3 and 4). Further, among those with mild disabilities, the medical needs were found to be high, while the rehabilitation needs were found to be high for those without a disability (i.e., who incurred slight or no extant impairments; Supplementary 2). These outcomes find corroboration in the literature, which asserted an increase in the possibility of experiencing unmet medical needs by workers when their physical activity is restricted [10]. Namely, for workers with mild cases of impairment, the need for medical examinations stemming from inconveniences caused by the injury (e.g., pain or injured areas) relates mostly to the prevention of recurrence and physical activity to work 8 h [35]. Moreover, workers with severe disabilities or damages that represented severe and moderate disabilities were shown to experience difficulties when returning to work, owing mostly to external factors and severe extant [27, 28, 30].

Regular/temporary workers who deemed their medical needs to have been met showed ORs for return to original work and return to work of 1.85 (1.27–2.69) and 1.54 (1.12–2.13), respectively (Table 3). Regarding regular/temporary workers, at the time of the panel study, the target population of the study comprised injured workers whose claims were closed in 2017, and the survey began in 2018 (between August and October)—as such, 1 year had passed since the termination of their recovery period, so they may have already returned to their original workplace. Regarding medical needs by worker status, a study demonstrated that temporary workers tend to experience unmet medical needs owing to financial burden, while regular workers tend to experience unmet medical needs owing to lack of time [11, 36]. Thus, the environments related to the provision of medical evaluations for the work-related injury could have influenced the present findings.

This study is useful in that the findings are representative of injured workers in South Korea since it used panel data organized by the Korea Workers’ Compensation and Welfare Service, which followed-up with injured workers for 2 years after the termination of their recovery period [4, 15, 25]. Furthermore, this study examined the relationship between unmet medical needs and return to work, providing findings that may be meaningful to the literature as the first study to analyze these variables in South Korean workers who suffered from work-related injuries.

### Study limitations

First, the operational definition of unmet medical needs was based on patients’ subjective evaluation, and the analyzed groups were categorized by arbitrary definitions established by researchers based on the survey questions. The drawback is that three questions in the research were defined through injured workers’ subjective evaluations, rather than through objective assessment. Subjective perceptions, however, are commonly used in survey-based studies as indexes for various constructs [9].

Second, at the time of the panel study, for workers who responded that they returned to work, the responses on their medical and rehabilitation needs may have been influenced by their work environments.

Third, based on prior research [25], it is presumed that recall bias among injured workers may have influenced the results of this study; this is because the present study processed data that were obtained through survey visits that were conducted during a specific period.

Fourth, a limitation of the outcome variable is that the survey is planned to be performed over 5 years; however, because this study was conducted midway through this 5-year period, only data from the first 2 years were available for analysis. Although the follow-up period was rather short, this study found that there were clear differences according to the type of return to work.

Finally, a limitation of the outcome variable is focused on first return to work outcome of paid work (excluding self-employment) across the two survey ways. This means that it does not fully capture the sequence in which it happened. Therefore, further research is needed when the secondary cohort survey is completed.

## Conclusions

For workers who sustained work-related injuries, providing medical accessibility and meeting rehabilitation needs were found to be important predictors of return to work after initial treatment. Specifically, workers with mild disabilities were more likely to return to work after their post-initial healthcare needs had been met and when the environment where they worked could satisfy their needs to return to work. Therefore, to increase the rates for return to work and provide a supportive workplace environment for work-related injury, victims, managers, policymakers, and many other stakeholders should aim to meet such workers’ medical and rehabilitation needs, even after their initial medical treatments have been completed.

## Supplementary Information

**Additional file 1: Supplementary Table 1**. Participants’ return to work status. **Supplementary Table 2**. Participants’ unmet medical needs and rehabilitation service needs by disability rating, industry, and work status.

## Data Availability

The data that support the findings of this study are available from the Labor Welfare Research Center of Korea Workers’ Compensation and Welfare Service. However, restrictions apply to the availability of such data, which were used under license for the current study, and so these are not publicly available. Nonetheless, data are available from the authors upon reasonable request and with permission of the Labor Welfare Research Center of Korea Workers’ Compensation and Welfare Service.
