# Supplementary material for: Satisfying medical and rehabilitation needs positively influences returning to work after a work-related injury: an analysis of national panel data from 2018 to 2019
Source: BMC Public Health. 2021 Nov 5;21:2017. doi: 10.1186/s12889-021-12064-1 (PMC8571869; doi:10.1186/s12889-021-12064-1)
Supplement: Supplementary file 1 — Additional file 1: Supplementary Table 1. Participants’ return to work status. Supplementary Table 2. Participants’ unmet medical needs and rehabilitation service needs by disability rating, industry, and work status. [file 12889_2021_12064_MOESM1_ESM.docx]

Supplementary Table 1. Participants’ return to work status

| Variables | | Non return to work | | Returned to original work | | | Reemployed | | p-value |
| --- | --- | --- | --- | --- | --- | --- | --- | --- | --- |
|  |  | N | % | N | | % | N | % |  |
| Total | | 662 | 28.9 | 683 | 29.8 | | 948 | 41.3 |  |
| Return to work status | |  |  |  |  | |  |  | <0.001 |
| Returned to original work → continued | |  |  | 532 | 23.2 | |  |  |  |
| Returned to original work → other | |  |  | 104 | 4.5 | |  |  |  |
| Reemployed → continued | |  |  |  |  | | 368 | 16.1 |  |
| Reemployed → other | |  |  |  |  | | 247 | 10.8 |  |
| Self-employed → continued | |  |  |  |  | | 58 | 2.5 |  |
| Self-employed → other | |  |  |  |  | | 7 | 0.3 |  |
| Unemployed, Economic inactivity→ Returned to original work |  | | | 47 | 2.1 | |  |  |  |
| Unemployed, Economic inactivity → Reemployed | | | |  |  | | 242 | 10.6 |  |
| Unemployed, Economic inactivity → Self-employed | | | |  |  | | 26 | 1.1 |  |
| Other | | 662 | 28.9 |  |  | |  |  |  |

Supplementary Table 2. Participants’ unmet medical needs and rehabilitation service needs by disability rating, industry, and work status

| Variables | Unmet medical needs | | | | p-value | Rehabilitation service needs | | | | p-value |
| --- | --- | --- | --- | --- | --- | --- | --- | --- | --- | --- |
|  | Yes | | No | |  | Yes | | No | |  |
|  | N | % | N | % |  | N | % | N | % |  |
| Total | 185 | 8.1 | 2108 | 91.9 |  | 1571 | 68.5 | 722 | 31.5 |  |
| Disability rating |  |  |  |  | 0.982 |  |  |  |  | <0.001 |
| Severe | 23 | 8.2 | 257 | 91.8 |  | 225 | 59.1 | 156 | 40.9 |  |
| Moderate | 46 | 8.0 | 529 | 92.0 |  | 535 | 64.8 | 291 | 35.2 |  |
| Mild | 84 | 8.3 | 934 | 91.8 |  | 358 | 70.2 | 152 | 29.8 |  |
| None | 32 | 7.6 | 388 | 92.4 |  | 453 | 78.7 | 123 | 21.4 |  |
| Industry |  |  |  |  | 0.301 |  |  |  |  | 0.147 |
| Manufacturing | 59 | 8.4 | 648 | 91.7 |  | 486 | 68.7 | 221 | 31.3 |  |
| Construction | 64 | 8.4 | 694 | 91.6 |  | 519 | 68.5 | 239 | 31.5 |  |
| Service | 17 | 5.4 | 299 | 94.6 |  | 231 | 73.1 | 85 | 26.9 |  |
| Other | 45 | 8.8 | 467 | 91.2 |  | 335 | 65.4 | 177 | 34.6 |  |
| Work status |  |  |  |  | 0.366 |  |  |  |  | 0.793 |
| Regular/temporary worker | 129 | 8.4 | 1401 | 91.6 |  | 1051 | 68.7 | 479 | 31.3 |  |
| Daily worker | 56 | 7.3 | 707 | 92.7 |  | 520 | 68.2 | 243 | 31.9 |  |
